# Supplementary material for: Diagnosis of Acute Myocarditis Using Texture-Based Cardiac Magnetic Resonance, with CINE Imaging as a Novel Tissue Characterization Technique
Source: Diagnostics (Basel). 2022 Dec 16;12(12):3187. doi: 10.3390/diagnostics12123187 (PMC9777125; doi:10.3390/diagnostics12123187)
Supplement: Supplementary file 1 [file diagnostics-12-03187-s001.zip › diagnostics-2038946-supplementary.pdf]

Supplementary Materials

Table S1. Complete list of all robust and significant texture features, with moderate to strong correlation with AM, and corresponding statistical data.

CMR	Texture Feature	CMR-Verified AM	Non CMR-Verified AM	ICC	*p*-Value	*R*
T2w	WavEnLL_s_2	16,400 ± 1360	19,100 ± 1600	0.85	0.002	-0.67
	WavEnLL_s_3	12,500 ± 1690	16,900 ± 3570	0.88	0.008	-0.62
	S_3__3_SumOfSqs	113 ± 8	103 ± 5	0.88	0.012	0.59
	S_2__2_SumOfSqs	111 ± 6	104 ± 4	0.81	0.012	0.59
	WavEnLL_s_4	8050 ± 1920	12,200 ± 3600	0.94	0.012	-0.58
	S_1_1_SumAverg	64.7 ± 0.8	63.8 ± 0.6	0.77	0.012	0.56
	S_2_2_SumAverg	65.2 ± 1.2	63.8 ± 0.9	0.89	0.019	0.54
	S_4__4_SumOfSqs	113 ± 9	102 ± 10	0.81	0.019	0.50
	Skewness	0.37 ± 0.38	-0.01 ± 0.3	0.89	0.031	0.49
	S_0_2_SumOfSqs	110 ± 5	106 ± 4	0.81	0.021	0.48
	S_0_1_SumAverg	64.5 ± 0.6	63.9 ± 0.5	0.80	0.021	0.46
LGE	S_0_2_SumVarnc	401 ± 46	306 ± 42	0.98	<0.001	0.73
	S_0_4_SumVarnc	376 ± 73	233 ± 65	0.96	<0.001	0.72
	S_0_5_SumVarnc	372 ± 82	228 ± 66	0.97	<0.001	0.69
	S_1__1_SumVarnc	411 ± 28	258 ± 29	0.95	<0.001	0.69
	S_0_1_SumVarnc	424 ± 24	379 ± 23	0.98	<0.001	0.68
	S_2_0_SumAverg	65.6 ± 0.9	63.6 ± 1.3	0.78	<0.001	0.68
	S_0_2_SumAverg	65.9 ± 1.1	63.6 ± 1.4	0.77	<0.001	0.68
	S_0_2_SumOfSqs	114 ± 6	102 ± 7	0.89	<0.001	0.66
	WavEnLL_s_2	17,700 ± 1540	21,200 ± 2320	0.91	<0.001	-0.66
	S_0_4_Correlat	0.57 ± 0.21	0.18 ± 0.23	0.98	<0.001	0.66
	S_0_5_Correlat	0.54 ± 0.20	0.19 ± 0.20	0.98	<0.001	0.66
	WavEnLH_s_3	360 ± 146	643 ± 178	0.99	<0.001	-0.66
	S_1_1_SumVarnc	413 ± 34	362 ± 26	0.98	<0.001	0.64
	WavEnLL_s_3	22,500 ± 4050	29,100 ± 3920	0.95	<0.001	-0.64
	WavEnLL_s_4	27,800 ± 5490	36,700 ± 5560	0.99	<0.001	-0.63
	S_0_2_Correlat	0.76 ± 0.13	0.50 ± 0.19	0.99	<0.001	0.62
	S_1__1_Correlat	0.85 ± 0.06	0.71 ± 0.11	0.98	0.001	0.62
	S_0_4_SumOfSqs	119 ± 10	97.6 ± 16.2	0.86	<0.001	0.62
	S_2_2_SumAverg	66.2 ± 1.2	63.9 ± 1.6	0.82	<0.001	0.62
	WavEnHH_s_2	64.3 ± 30.2	141 ± 63	0.99	<0.001	-0.61
	WavEnLH_s_2	214 ± 101	460 ± 202	0.98	<0.001	-0.61
	WavEnLH_s_1	71.9 ± 35.1	159 ± 72	0.98	0.001	-0.61
	S_0_1_Correlat	0.92 ± 0.05	0.83 ± 0.07	0.99	<0.001	0.61
	S_2_2_SumOfSqs	117 ± 8	106 ± 7	0.94	<0.001	0.61
	S_0_5_SumOfSqs	120 ± 14	95.4 ± 18.4	0.91	<0.001	0.61
	S_0_4_SumAverg	67.3 ± 2.7	63.7 ± 2.1	0.89	<0.001	0.60
	S_1_0_SumVarnc	423 ± 23	391 ± 21	0.96	<0.001	0.60
	S_0_1_Contrast	17.8 ± 9.4	36.4 ± 15.7	0.99	0.001	-0.59
	S_1__1_Contrast	33.5 ± 11.3	61.8 ± 25.4	0.98	0.002	-0.59
	S_2_2_SumVarnc	373 ± 60	296 ± 48	0.99	0.001	0.59
	S_2_0_SumVarnc	386 ± 37	332 ± 39	0.97	0.001	0.58
	S_0_2_Contrast	53.8 ± 27.4	102 ± 40	0.99	0.002	-0.57
	WavEnHL_s_1	84.7 ± 25.3	150 ± 62	0.99	0.003	-0.57

WavEnHH_s_1	9.18 ± 5.40	27.2 ± 17.8	0.97	0.002	-0.57
S_1_1_SumOfSqs	113 ± 5	105 ± 6	0.96	0.001	0.57
S_0_5_SumEntrp	1.80 ± 0.04	1.70 ± 0.09	0.95	<0.001	0.56
S_0_5_SumAverg	67.9 ± 3.5	63.8 ± 2.4	0.91	0.002	0.56
S_0_1_DifVarnc	8.03 ± 4.04	15.7 ± 7.1	0.99	0.001	-0.56
S_0_4_SumEntrp	1.80 ± 0.04	1.71 ± 0.09	0.95	<0.001	0.55
S_1__1_DifVarnc	15.1 ± 4.8	26.1 ± 10.8	0.96	0.002	-0.55
Sigma	0.22 ± 0.05	0.33 ± 0.11	0.99	0.002	-0.55
S_0_2_DifVarnc	23.9 ± 11.7	42.4 ± 17.1	0.99	0.002	-0.54
S_0_4_Contrast	100 ± 42	157 ± 49	0.98	0.002	-0.53
S_0_1_InvDfMom	0.32 ± 0.06	0.24 ± 0.07	0.99	0.001	0.53
S_5_5_Contrast	149 ± 39	204 ± 50	0.86	0.002	-0.53
S_0_4_InvDfMom	0.15 ± 0.03	0.11 ± 0.03	0.95	0.001	0.52
S_0_2_InvDfMom	0.21 ± 0.04	0.15 ± 0.05	0.99	0.001	0.52
WavEnHL_s_2	275 ± 100	436 ± 160	0.97	0.003	-0.52
S_0_1_SumOfSqs	110 ± 4	104 ± 6	0.95	0.002	0.52
S_5_5_Correlat	0.35 ± 0.21	0.11 ± 0.19	0.95	0.002	0.51
GrMean	2.24 ± 0.39	2.85 ± 0.63	0.98	0.003	-0.50
S_1_1_Correlat	0.83 ± 0.07	0.72 ± 0.11	<1.00	0.003	0.50
S_1_0_Correlat	0.90 ± 0.03	0.84 ± 0.07	0.98	0.008	0.50
S_4__4_Correlat	0.39 ± 0.18	0.20 ± 0.16	0.93	0.003	0.50
S_0_1_DifEntrp	0.94 ± 0.10	1.06 ± 0.11	0.99	0.002	-0.50
S_2_0_SumOfSqs	114 ± 6	107 ± 6	0.89	0.006	0.50
S_0_2_DifEntrp	1.16 ± 0.10	1.28 ± 0.11	0.98	0.003	-0.49
S_0_5_InvDfMom	0.14 ± 0.03	0.11 ± 0.03	0.94	0.002	0.49
S_4__4_InvDfMom	0.14 ± 0.03	0.11 ± 0.02	0.90	0.004	0.49
S_1__1_DifEntrp	1.07 ± 0.07	1.17 ± 0.10	0.98	0.002	-0.49
WavEnLH_s_4	384 ± 149	540 ± 132	0.99	0.003	-0.49
S_1__1_InvDfMom	0.24 ± 0.05	0.19 ± 0.05	0.99	0.002	0.48
S_0_5_Contrast	108 ± 38	153 ± 46	0.97	0.005	-0.48
S_4_4_Correlat	0.40 ± 0.20	0.19 ± 0.19	0.97	0.004	0.48
S_4_4_Contrast	138 ± 38	182 ± 45	0.92	0.006	-0.47
S_5_5_DifVarnc	60.4 ± 14.5	78.6 ± 19.4	0.87	0.007	-0.47
S_1_0_Contrast	21.6 ± 5.4	33.5 ± 15.0	0.98	0.008	-0.47
S_1_1_Contrast	37.2 ± 14.9	58.2 ± 24.0	0.99	0.006	-0.47
S_2_2_Correlat	0.59 ± 0.17	0.39 ± 0.20	0.99	0.005	0.47
S_5__5_Correlat	0.33 ± 0.19	0.15 ± 0.16	0.91	0.005	0.46
S_0_4_DifVarnc	43.7 ± 18.3	63.2 ± 19.8	0.98	0.007	-0.46
S_1__1_SumOfSqs	111 ± 5	105 ± 7	0.90	0.008	0.45
S_2_0_Correlat	0.69 ± 0.09	0.56 ± 0.17	0.97	0.016	0.45
S_1_1_InvDfMom	0.24 ± 0.04	0.19 ± 0.06	0.99	0.002	0.45
S_0_4_DifEntrp	1.29 ± 0.08	1.37 ± 0.08	0.98	0.007	-0.44
S_1_0_SumOfSqs	111 ± 5	106 ± 6	0.94	0.007	0.44
S_4__4_Contrast	131 ± 36	169 ± 44	0.86	0.017	-0.43
S_2_2_InvDfMom	0.16 ± 0.03	0.12 ± 0.04	<1.00	0.003	0.42
S_1_0_DifVarnc	9.84 ± 2.29	13.9 ± 6.0	0.96	0.019	-0.41
S_5__5_InvDfMom	0.13 ± 0.03	0.10 ± 0.03	0.89	0.025	0.41
S_2_0_Contrast	69.3 ± 16.0	95.1 ± 38.0	0.96	0.020	-0.41

	S_2_2_Contrast	95.1 ± 33.2	129 ± 43	0.98	0.015	-0.40
	S_4_4_SumVarnc	334 ± 84	269 ± 64	0.99	0.016	0.40
CINE	S_0_1_Correlat	0.84 ± 0.06	0.72 ± 0.07	0.98	<0.001	0.70
	S_0_2_Correlat	0.61 ± 0.15	0.32 ± 0.15	0.99	<0.001	0.70
	S_0_2_SumVarnc	359 ± 50	274 ± 39	0.98	<0.001	0.69
	S_0_1_Contrast	34.0 ± 13.2	59.9 ± 13.9	0.97	<0.001	-0.68
	S_0_2_Contrast	85.3 ± 31.8	141 ± 28	0.97	<0.001	-0.68
	S_1__1_Correlat	0.73 ± 0.10	0.54 ± 0.11	0.98	<0.001	0.67
	S_1__1_Contrast	57.8 ± 20.3	94.9 ± 21.5	0.98	<0.001	-0.66
	S_0_1_SumVarnc	405 ± 25	362 ± 25	0.89	<0.001	0.66
	S_0_3_Correlat	0.51 ± 0.19	0.20 ± 0.17	<1.00	<0.001	0.66
	Sigma	0.35 ± 0.08	0.48 ± 0.08	0.98	<0.001	-0.65
	S_0_3_SumVarnc	344 ± 61	252 ± 47	0.98	<0.001	0.65
	S_0_1_DifVarnc	15.4 ± 6.2	25.9 ± 6.5	0.97	<0.001	-0.64
	S_0_5_Correlat	0.51 ± 0.19	0.20 ± 0.18	0.97	<0.001	0.64
	S_1__1_SumVarnc	376 ± 40	317 ± 32	0.94	<0.001	0.64
	S_0_2_DifVarnc	35.3 ± 13.5	56.8 ± 12.9	0.96	<0.001	-0.63
	GrMean	2.74 ± 0.56	3.48 ± 0.33	0.99	<0.001	-0.63
	S_0_4_Correlat	0.51 ± 0.19	0.22 ± 0.18	0.99	<0.001	0.63
	S_1__1_DifVarnc	25.2 ± 8.71	39.7 ± 9.57	0.97	<0.001	-0.62
	S_0_2_SumEntrp	1.83 ± 0.03	1.75 ± 0.06	0.90	<0.001	0.62
	S_0_3_Contrast	109 ± 40	167 ± 34	0.98	<0.001	-0.62
	S_0_3_SumEntrp	1.81 ± 0.03	1.73 ± 0.07	0.87	<0.001	0.62
	WavEnLH_s_1	192 ± 75	336 ± 108	0.97	<0.001	-0.62
	S_1_1_Correlat	0.72 ± 0.11	0.57 ± 0.08	0.99	<0.001	0.61
	S_1_1_Contrast	59.6 ± 23.4	91.1 ± 17.6	0.98	<0.001	-0.61
	WavEnHH_s_2	174 ± 54	261 ± 62	0.97	<0.001	-0.61
	S_0_4_SumVarnc	354 ± 67	261 ± 55	0.94	<0.001	0.60
	S_2__2_Correlat	0.50 ± 0.20	0.22 ± 0.18	0.99	<0.001	0.60
	S_0_5_SumVarnc	361 ± 79	259 ± 60	0.88	<0.001	0.59
	S_0_3_DifVarnc	43.3 ± 16.4	65.2 ± 14.5	0.97	<0.001	-0.58
	S_1__1_SumEntrp	1.85 ± 0.02	1.78 ± 0.06	0.91	0.001	0.58
	S_2_0_SumEntrp	1.81 ± 0.03	1.75 ± 0.05	0.91	0.001	0.58
	S_2_2_Correlat	0.48 ± 0.21	0.24 ± 0.12	<1.00	0.001	0.57
	S_2_2_Contrast	110 ± 43	164 ± 35	0.97	0.001	-0.57
	S_0_1_InvDfMom	0.24 ± 0.05	0.18 ± 0.02	0.99	<0.001	0.57
	S_2__2_SumEntrp	1.81 ± 0.03	1.73 ± 0.07	0.90	0.001	0.57
	S_0_4_SumEntrp	1.81 ± 0.03	1.72 ± 0.08	0.80	<0.001	0.57
	S_0_4_Contrast	111 ± 42	162 ± 32	0.99	0.001	-0.57
	S_2__2_SumVarnc	329 ± 67	246 ± 54	0.98	<0.001	0.57
	S_0_5_DifVarnc	44.5 ± 16.1	65.6 ± 14.8	0.99	<0.001	-0.57
	S_1_0_Correlat	0.83 ± 0.07	0.73 ± 0.07	0.99	<0.001	0.57
	S_0_5_Contrast	115 ± 43	168 ± 35	0.99	<0.001	-0.57
	S_0_5_SumEntrp	1.8 ± 0.03	1.71 ± 0.09	0.84	<0.001	0.57
	S_1_1_DifVarnc	25.0 ± 10.0	37.5 ± 8.37	0.98	0.001	-0.56
	S_1_1_SumEntrp	1.84 ± 0.02	1.78 ± 0.06	0.94	0.002	0.56
	S_0_1_DifEntrp	1.07 ± 0.09	1.17 ± 0.06	0.99	<0.001	-0.56
	GrVariance	2.95 ± 1.05	4.37 ± 1.08	0.98	0.002	-0.56

S_2_2_DifVarnc	43.3 ± 16.1	64.8 ± 16.1	0.97	<0.001	-0.56
S_0_2_InvDfMom	0.15 ± 0.04	0.12 ± 0.01	0.99	<0.001	0.56
S_1_0_Contrast	36.7 ± 14.0	55.9 ± 14.8	0.99	<0.001	-0.56
S_2__2_Contrast	108 ± 39	155 ± 33	0.98	0.001	-0.56
S_2_0_Correlat	0.55 ± 0.18	0.34 ± 0.14	0.99	0.002	0.55
S_0_2_DifEntrp	1.26 ± 0.09	1.35 ± 0.04	<1.00	<0.001	-0.55
S_1_1_SumVarnc	372 ± 34	333 ± 26	0.92	0.001	0.55
S_2_0_Contrast	94.1 ± 36.6	137 ± 30	0.98	0.001	-0.54
S_1__1_DifEntrp	1.18 ± 0.08	1.27 ± 0.06	0.99	<0.001	-0.54
S_2__2_InvDfMom	0.14 ± 0.03	0.11 ± 0.01	0.99	<0.001	0.54
S_0_2_SumOfSqs	111 ± 6	104 ± 5	0.78	0.002	0.53
S_2_2_SumEntrp	1.79 ± 0.03	1.74 ± 0.05	0.97	0.006	0.53
S_1__1_InvDfMom	0.19 ± 0.04	0.14 ± 0.02	0.99	<0.001	0.53
S_0_4_DifVarnc	43.7 ± 15.8	62.7 ± 14.8	0.98	0.002	-0.53
S_2_0_SumVarnc	329 ± 47	277 ± 36	0.96	0.002	0.53
S_0_1_SumEntrp	1.86 ± 0.02	1.80 ± 0.07	0.95	0.003	0.53
S_1_0_DifVarnc	15.8 ± 6.0	23.4 ± 6.4	0.97	0.001	-0.53
WavEnHH_s_1	37.7 ± 18.2	82.5 ± 48.4	0.98	<0.001	-0.53
Perc_90_	84.2 ± 34.5	51.9 ± 14.7	<1.00	0.003	0.52
S_2_0_DifVarnc	38.3 ± 14.4	54.5 ± 12.1	0.96	0.002	-0.52
WavEnHL_s_1	202 ± 62	296 ± 91	0.91	0.002	-0.52
S_3_3_Contrast	121 ± 50	178 ± 45	0.96	0.002	-0.52
S_1_0_SumVarnc	394 ± 27	364 ± 24	0.87	0.003	0.52
S_3_0_SumEntrp	1.78 ± 0.04	1.73 ± 0.06	0.93	0.002	0.52
S_3_3_Correlat	0.42 ± 0.23	0.21 ± 0.11	0.99	0.002	0.51
S_3_3_InvDfMom	0.12 ± 0.03	0.10 ± 0.01	0.98	0.002	0.50
Perc_99_	101 ± 43	63.0 ± 18.8	0.99	0.003	0.50
Mean	65.6 ± 27.0	41.9 ± 10.6	0.99	0.008	0.50
S_3_3_DifVarnc	46.9 ± 18.6	67.3 ± 16.7	0.94	0.002	-0.50
S_1_1_InvDfMom	0.18 ± 0.04	0.14 ± 0.02	0.99	0.003	0.50
S_2__2_DifVarnc	44.2 ± 15.8	62.2 ± 15.7	0.97	0.004	-0.50
Perc_50_	64.2 ± 26.4	41.4 ± 10.3	<1.00	0.009	0.50
WavEnLH_s_2	432 ± 107	586 ± 158	0.96	0.006	-0.50
S_3__3_SumEntrp	1.79 ± 0.04	1.71 ± 0.09	0.76	0.005	0.50
S_0_2_SumAverg	64.4 ± 0.98	63.1 ± 1.32	0.83	0.005	0.50
S_1_0_SumEntrp	1.86 ± 0.02	1.80 ± 0.07	0.95	0.005	0.49
S_2_2_SumVarnc	313 ± 52	267 ± 27	0.97	0.007	0.49
S_3__3_Correlat	0.45 ± 0.23	0.22 ± 0.18	0.99	0.003	0.49
S_0_3_SumOfSqs	113 ± 9	105 ± 7	0.81	0.007	0.49
S_1_1_DifEntrp	1.18 ± 0.09	1.26 ± 0.05	0.99	0.003	-0.48
S_3__3_SumVarnc	328 ± 85	243 ± 71	0.97	0.003	0.48
S_1_0_InvDfMom	0.22 ± 0.05	0.18 ± 0.03	0.99	0.005	0.48
Variance	222 ± 178	75 ± 74	<1.00	0.003	0.48
S_4__4_SumEntrp	1.77 ± 0.05	1.67 ± 0.12	0.81	0.007	0.48
S_3_0_Correlat	0.40 ± 0.24	0.18 ± 0.16	0.98	0.007	0.48
Perc_01_	38.7 ± 17.6	24.2 ± 7.3	0.96	0.032	0.47
S_0_4_InvDfMom	0.13 ± 0.03	0.10 ± 0.01	0.98	0.005	0.47
Perc_10_	49.4 ± 21.6	32.2 ± 7.9	0.99	0.047	0.47

S_1__1_SumAverg	64.2 ± 0.8	63.3 ± 1.0	0.83	0.006	0.47
S_1__1_SumOfSqs	108 ± 6	103 ± 5	0.76	0.008	0.47
S_0_3_SumAverg	64.4 ± 1.5	62.8 ± 1.8	0.86	0.008	0.46
S_5_5_Contrast	142 ± 55	195 ± 46	0.91	0.009	-0.46
S_3_3_SumEntrp	1.78 ± 0.04	1.73 ± 0.05	0.86	0.009	0.46
S_5__5_SumEntrp	1.74 ± 0.06	1.65 ± 0.11	0.81	0.009	0.46
S_0_3_DifEntrp	1.31 ± 0.09	1.38 ± 0.05	<1.00	0.005	-0.46
S_0_3_InvDfMom	0.13 ± 0.03	0.11 ± 0.01	0.99	0.003	0.46
S_4_4_Contrast	135 ± 57	186 ± 42	0.93	0.009	-0.46
S_0_1_SumAverg	64.4 ± 0.6	63.5 ± 1.0	0.79	0.008	0.45
S_2_2_DifEntrp	1.32 ± 0.09	1.38 ± 0.04	0.99	0.007	-0.45
S_4__4_Entropy	2.66 ± 0.10	2.49 ± 0.21	0.76	0.014	0.45
S_4__4_Correlat	0.40 ± 0.26	0.17 ± 0.20	0.99	0.010	0.45
S_3_0_SumVarnc	293 ± 54	246 ± 40	0.97	0.010	0.45
S_3_0_Contrast	126 ± 51	170 ± 37	0.94	0.007	-0.45
S_0_4_SumOfSqs	116 ± 12	106 ± 9	0.79	0.018	0.45
S_2__2_SumAverg	64.0 ± 1.1	62.8 ± 1.3	0.80	0.007	0.44
S_0_5_Entropy	2.69 ± 0.09	2.54 ± 0.21	0.78	0.014	0.44
S_4__4_SumVarnc	322 ± 105	230 ± 83	0.99	0.010	0.44
S_3__3_SumAverg	63.9 ± 1.76	62.3 ± 1.6	0.84	0.014	0.44
S_5__5_SumVarnc	318 ± 115	220 ± 86	0.99	0.009	0.44
S_0_3_Entropy	2.75 ± 0.09	2.62 ± 0.18	0.82	0.023	0.43
S_2__2_SumOfSqs	109 ± 8	100 ± 10	0.87	0.009	0.43
S_0_4_AngScMom	0.002 ± 0.0006	0.004 ± 0.002	0.76	0.026	-0.43
S_5__5_Correlat	0.37 ± 0.27	0.15 ± 0.21	0.98	0.010	0.43
S_0_5_AngScMom	0.003 ± 0.0006	0.004 ± 0.002	0.77	0.025	-0.43
S_0_5_DifEntrp	1.32 ± 0.08	1.38 ± 0.05	0.98	0.009	-0.43
S_0_3_AngScMom	0.002 ± 0.0007	0.003 ± 0.001	0.88	0.036	-0.42
S_4_0_SumEntrp	1.77 ± 0.04	1.72 ± 0.05	0.89	0.010	0.42
S_2__2_Entropy	2.75 ± 0.09	2.62 ± 0.18	0.84	0.029	0.42
S_3_0_DifVarnc	50.1 ± 19.3	65.8 ± 14.6	0.93	0.011	-0.42
GrNonZeros	0.95 ± 0.03	0.97 ± 0.009	0.96	0.002	-0.42
S_1_0_DifEntrp	1.08 ± 0.09	1.15 ± 0.07	0.99	0.015	-0.42
S_5__5_SumOfSqs	113 ± 20	93.1 ± 23.8	0.96	0.032	0.41
S_2_2_InvDfMom	0.13 ± 0.03	0.11 ± 0.01	0.99	0.010	0.41
S_3_3_DifEntrp	1.33 ± 0.09	1.39 ± 0.05	0.96	0.013	-0.41
S_2_0_DifEntrp	1.28 ± 0.09	1.34 ± 0.06	0.99	0.013	-0.41
S_2_0_InvDfMom	0.15 ± 0.04	0.12 ± 0.02	0.99	0.018	0.41
S_4_4_DifVarnc	53.2 ± 22.6	70.3 ± 15.8	0.94	0.019	-0.40
S_0_5_SumOfSqs	119 ± 16	107 ± 12	0.77	0.027	0.40
S_2_2_AngScMom	0.002 ± 0.0006	0.003 ± 0.001	0.81	0.041	-0.40
S_3__3_AngScMom	0.003 ± 0.0008	0.004 ± 0.002	0.83	0.019	-0.40

AM: acute myocarditis; CMR: cardiac magnetic resonance; ICC: intraclass correlation coefficient; LGE: late gadolinium enhancement; R: Pearson's correlation coefficients; T2w: T2-weighted.
